# Assessing the Effectiveness of mHealth Interventions for Diabetes and Hypertension Management in Africa: Systematic Review and Meta-Analysis

**DOI:** 10.2196/43742

**Published:** 2023-08-29

**Authors:** Pearl Aovare, Kasim Abdulai, Amos Laar, Eva L van der Linden, Nicolaas Moens, Edo Richard, Eric P Moll van Charante, Charles Agyemang

**Affiliations:** 1 Department of Public & Occupational Health, University of Amsterdam, Amsterdam, Netherlands; 2 Department of Clinical Nutrition and Dietetics, University of Cape Coast, Cape Coast, Ghana; 3 Department of Population, Family and Reproductive Health, University of Ghana, Accra, Ghana; 4 Department of Vascular Medicine, University of Amsterdam, Amsterdam, Netherlands; 5 Department of Economics, eHealth, and Digital Transformation, Vrije Universiteit Amsterdam, Amsterdam, Netherlands; 6 Department of Neurology, University of Amsterdam, Amsterdam, Netherlands; 7 Department of Neurology, Donders Institute for Brain, Behaviour and Cognition, Radboud University Medical Centre, Nijmegen, Netherlands; 8 Department of General Practice, University of Amsterdam, Amsterdam, Netherlands

**Keywords:** mobile health, interventions, diabetes, blood sugar, hypertension, management, effectiveness, chronic disease, Africa, blood pressure, glycemic, intervention, mHealth, efficiency, resource

## Abstract

**Background:**

Mobile health (mHealth) interventions are effective in improving chronic disease management, mainly in high-income countries. However, less is known about the efficacy of mHealth interventions for the reduction of cardiovascular risk factors, including for hypertension and diabetes, which are rapidly increasing in low- and middle-income countries.

**Objective:**

This study aimed to assess the efficacy of mHealth interventions for diabetes and hypertension management in Africa.

**Methods:**

We searched PubMed, Cochrane Library, Google Scholar, African Journals Online, and Web of Science for relevant studies published from inception to July 2022. The main outcomes of interest were changes in hemoglobin A_1c_ (HbA_1c_), systolic blood pressure, and diastolic blood pressure. The random or fixed effect model was used for the meta-analysis, and the *I*^2^ statistic was used to gauge study heterogeneity. *Z* tests and *P* values were used to evaluate the effect of mHealth interventions on HbA_1c_ and blood pressure levels.

**Results:**

This review included 7 studies (randomized controlled trials) with a total of 2249 participants. Two studies assessed the effect of mHealth on glycemic control, and 5 studies assessed the effect of mHealth on blood pressure control. The use of mHealth interventions was not associated with significant reductions in HbA_1c_ levels (weighted mean difference [WMD] 0.20, 95% CI −0.40 to 0.80; *P*=.51) among patients with diabetes and systolic blood pressure (WMD −1.39, 95% CI −4.46 to 1.68; *P*=.37) and diastolic blood pressure (WMD 0.36, 95% CI −1.37 to 2.05; *P*=.69) among patients with hypertension. After conducting sensitivity analyses using the leave-one-out method, the Kingue et al study had an impact on the intervention, resulting in a 2 mm Hg reduction in systolic blood pressure (WMD −2.22, 95% CI −3.94 to −0.60; *P*=.01) but was nonsignificant for diastolic blood pressure and HbA_1c_ levels after omitting the study.

**Conclusions:**

Our review provided no conclusive evidence for the effectiveness of mHealth interventions in reducing blood pressure and glycemic control in Africa among persons with diabetes and hypertension. To confirm these findings, larger randomized controlled trials are required.

## Introduction

Managing chronic diseases often calls for a long-term care strategy [1]. Diabetes and hypertension remain two of the most common chronic conditions globally, resulting in the highest health care resource use and mortality [2-4]. Type 2 diabetes prevalence has become a substantial health issue, especially in African regions where type 2 diabetes is predicted to increase at the quickest rate (129%) in the world by 2045 [5-7]. Similarly, hypertension remains a major public health challenge among older adults in the African region, with an estimated pooled prevalence of 30.8% in Africa and between 30% and 31.1% in sub-Saharan Africa [8]. Poor blood pressure control among persons with hypertension is thought to involve intricate interactions between patients, health care providers, and socioeconomic variables [9]. Medication adherence has also been identified as one of the critical disease management issues, especially in enhancing life quality, health outcomes, and access to affordable health care worldwide [10,11].

Disease management programs using mobile health (mHealth) are promising emerging strategies to help patients self-manage their conditions (eg, measuring their blood pressure and sugar levels with remote professional support when needed [7,12]). mHealth is a medical and public health practice supported by portable electronic devices such as cell phones, patient monitoring devices, personal digital assistants, and other wireless gadgets [13]. This includes the use of phones and remote monitoring devices in health care and public health practice for communication, data collection, patient monitoring, and education, and to facilitate adherence to chronic disease management [14,15]. mHealth devices can improve service delivery and impact patient outcomes [15].

Previous studies in some low- and middle-income countries have assessed the application of mHealth as a tool to increase drug compliance in patients with a range of long-term illnesses, including diabetes, chronic obstructive pulmonary disease, and HIV infection [11,16]. Although several African countries are still in the pilot and development stages, an increasing number of mHealth apps have been put into use in clinical care settings [17]. The majority of small-scale pilot or feasibility mHealth intervention studies in Africa have been based on SMS text messaging systems to improve disease management [17,18].

Most individuals now possess mobile phones, and there are over 5.3 billion subscribers to mobile services worldwide, 67% of the world’s population [19,20]. There will be 400 million more new mobile service customers between now and 2025, the majority of whom will come from Asia Pacific and sub-Saharan Africa, increasing the total number of subscribers to 5.7 billion (70% of the global population) [21]. There have been individual studies in Africa about mHealth interventions on disease management [22], although the data on the efficacy of mHealth in the management of diabetes and hypertension in Africa are limited and have not yet been systematically evaluated. Therefore, this systematic review assessed the effectiveness of mHealth interventions on blood pressure control among patients with hypertension and glycemic control among patients with diabetes in Africa. The findings of this paper will guide improvements to the adoption of mHealth for the management of diabetes and hypertension in African countries.

## Methods

This systematic review was conducted following the PRISMA (Preferred Reporting Items for Systematic Reviews and Meta-Analyses) guidelines [23] (Multimedia Appendix 1). The protocol was registered in PROSPERO (CRD42021230642).

### Search Strategy

PubMed, Cochrane Library, Google Scholar, African Journals Online, and Web of Science were searched for relevant studies published from inception to July 2022, with assistance from a clinical librarian. The full search strategies, common Medical Subject Headings (MeSH), and search terms used across databases are available in Table S1 in Multimedia Appendix 2. The reference lists of the included studies were hand-searched to identify additional relevant studies.

### Study Selection

Two independent authors (PA and KA) manually assessed and screened studies for both the titles and abstracts as well as full-text articles using an Excel sheet (Microsoft Corporation). Disagreements were resolved by consensus with a third author (CA) as necessary. This was performed in three stages as follows. First, PA screened the titles of all papers to determine their relevance. KA performed a cross-check by screening 20% of the titles excluded by the first reviewer, and it was confirmed that none of the titles screened by the second reviewer met the inclusion criteria. Second, abstracts of the papers selected after the title screening stage were again screened by PA and KA following the same procedure as described in step one. Finally, the full texts of potentially relevant papers were retrieved and evaluated by PA and KA independently to ascertain their relevance and usefulness to the review. Disagreements were settled through dialogue with CA to reach an agreement. Duplicates were also identified using EndNote reference manager (version x9; Clarivate).

### Inclusion Criteria

We included studies that met the following criteria: the patients had hypertension or diabetes and were 18 years or older; the patients had received treatment at a selected health care setting; the intervention included an mHealth component; the results included target values of hemoglobin A_1c_ (HbA_1c_), systolic blood pressure, or diastolic blood pressure; the studies were randomized controlled trials (RCTs); the articles were written in English; and the studies were conducted in hospitals and primary health centers.

### Exclusion Criteria

We excluded studies in which the full text was not available after attempts to contact the author, the research participants were pregnant women or a specific patient population (eg, patients with cancer), the results did not describe primary outcomes, the primary intervention did not use mHealth devices, or the articles were unpublished manuscripts or conference abstracts.

### Risk of Bias Assessment

The quality of each study was assessed using a 28-point scoring system as adopted from the Downs and Black checklist [24]. The included studies focused on the following items for assessment: items 1 through 10 evaluated whether the information provided was adequate for the reader to make an objective assessment of the study’s findings; items 11 through 13 evaluated external validity, which examined the extent to which study findings could be applied to the population from which the study participants were drawn; items 14 through 20 assessed possible bias, which focused on biases in the assessment of the intervention and the result; and items 21 through 26 assessed confounding, which focused on biases in the research participant selection. To determine if neutral research results may be the result of chance or insufficient power, item 27 evaluated the study’s power (Table 1).

**Table 1.** Downs and Black quality assessment.

Studies	Information based on study findings score (questions 1-10)	External validity score (questions 11-13)	Potential bias score (questions 14-20)	Confounding score (questions 21-26)	Power of study score (question 27)	Total score (maximum score of 27)	Quality as per the cutoff described
Abaza and Marschollek [25], 2017	10	3	6	5	0	24	Good
Adjei et al [26], 2015	10	3	5	5	0	23	Good
Asante et al [27], 2020	10	3	4	5	0	22	Good
Bobrow et al [28], 2016	10	3	5	6	0	24	Good
Kingue et al [29], 2013	10	3	5	6	0	24	Good
Owolabi et al [30], 2019	10	3	6	5	0	24	Good
Sarfo et al [31], 2019	10	3	6	6	0	25	Good

### Data Extraction

Two authors (PA and KA) independently extracted the following study characteristics from each included article using a tested extraction form: first author, year of publication, mean age, the country where the study was conducted, the participant (patient with diabetes or hypertension), mHealth location (primary care setting, hospital, clinic, etc), condition (diabetes/hypertension), sample size, mHealth intervention, study design, and outcome of the intervention.

### Data Synthesis and Analysis

The data for the primary outcomes (HbA_1c_, systolic blood pressure, and diastolic blood pressure) were analyzed separately using random or fixed effects models with a weighted mean difference (WMD) in ReviewManager (version 5.4; The Cochrane Collaboration) [32]. The *I*^2^ statistic was calculated to measure the percentage of variation across trials due to heterogeneity, with values of <50% and ≥50% indicating low and high levels of heterogeneity, respectively. The WMD for blood pressure and HbA_1c_ between the intervention and control and *Z* tests were used to compare groups, and *P*<.05 was regarded as statistically significant. We checked publication bias subjectively by funnel plots and objectively by Begg and Egger tests using Stata version 16 (StataCorp). Begg and Egger tests with *P*<.05 were considered to have signiﬁcant publication bias.

## Results

### Study Selection

**Figure 1.**
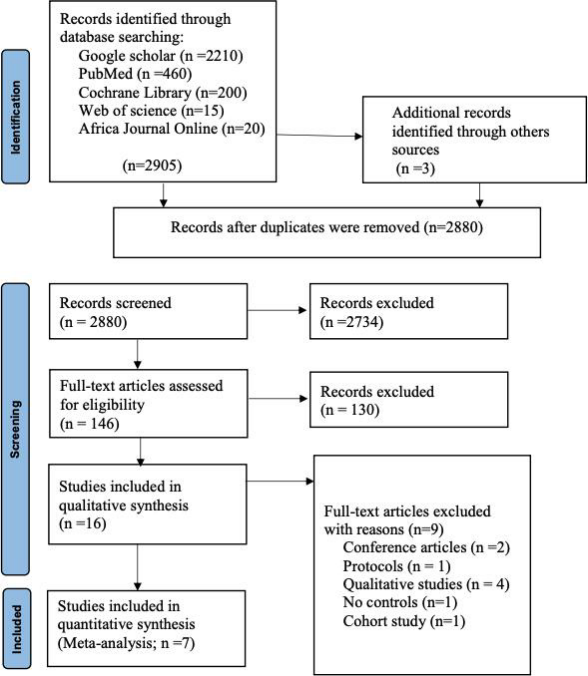
PRISMA (Preferred Reporting Items for Systematic Reviews and Meta-Analyses) flow diagram of evidence search and selection.

We identified 2908 records from our search; authors screened 2880 titles and abstracts after duplicates were removed. In total, 7 studies (RCTs) [25-29,31] were considered eligible for inclusion. The reasons for excluding a study are provided in Figure 1.

### Study Characteristics

The characteristics of the studies are shown in Tables 2 and 3. Of the 2249 participants, 1824 (54.1%) were female, the mean age ranged from 51.2 to 60.6 years, and the sample sizes ranged from 60 to 1372 participants. The studies were conducted at hospitals and primary health centers in the following countries: Ghana (n=3), South Africa (n=2), Egypt (n=1), and Cameroon (n=1). Five studies [26-29,31] reported the apps’ effectiveness in controlling blood pressure among patients with hypertension (Table 4), while 2 studies [25,27] reported the effect on HbA_1c_ levels in patients with diabetes (Table 5). Of the 7 included studies, 2 articles had more than one intervention group with the same outcome measure [25,28].

**Table 2.** Study characteristics.

Studies	Study design	Country	Gender (male/female; %)	Condition	Type of patient	Patients who received treatment (%)	mHealth^a^ study settings
Abaza and Marschollek [25], 2017	RCT^b^	Egypt	47/53	Diabetes	Adult with diabetes	—^c^	Clinic
Adjei et al [26], 2015	RCT	Ghana	44/56	Diabetes	Adult with diabetes	—	Health care center
Asante et al [27], 2020	RCT	Ghana	27/73	Diabetes	Adults with type 2 diabetes	66.3	Health center
Bobrow et al [28], 2016	RCT	South Africa	56/44	Hypertension	Adults with hypertension	50	Primary care clinic
Kingue et al [29], 2013	RCT	Cameroon	35/65	Hypertension	Young adults with hypertension	73.3	Clinic
Owolabi et al [30], 2019	RCT	South Africa	84/16	Diabetes	Adult with diabetes	75	Primary health care centers
Sarfo et al [31], 2019	RCT	Ghana	65/35	Hypertension	Adults with hypertension	13.3	Clinic

a mHealth: mobile health.

b RCT: randomized controlled trial.

c Not available.

**Table 3.** Study intervention and control description.

Studies	Intervention type (duration)	Intervention group	Control group
Abaza and Marschollek [25], 2017	SMS text messaging (3 mo)	Patients received daily messages and weekly reminders addressing various diabetes care categories.	The control group did not receive SMS text messages but received paper-based educational material.
Adjei et al [26], 2015	Electronic reminders (6 mo)	The intervention group was given electronic reminders for their clinical appointments, and their physicians were prompted for abnormal laboratory results [33] for 6 months.	Patients received only the usual care.
Asante et al [27], 2020	Mobile phone calls (3 mo)	The mobile phone call intervention was delivered by nurses in addition to care as usual over 12 weeks. The intervention group received up to 16 mobile phone calls (mean duration 12 min) from a diabetes specialist nurse in addition to their care as usual.	The control group received only care as usual.
Bobrow et al [28], 2016	SMS text messaging (6 and 12 mo)	SMS text messages were delivered automatically via an open source, web-based electronic medical record system. Texts were sent for 1 year from enrollment. Blood pressure measurements were collected from participants as they attended their routine clinic visits. The delivery of texts was automatically tracked, and if undelivered, a research assistant that was blinded to group allocation would contact the number of a friend or relative to obtain a new mobile phone number	The usual care group continued to receive care from the clinic and some form of written information about hypertension and healthy living, but no personalized SMS text messages were sent.
Kingue et al [29], 2013	Mobile phone calls (24 wk)	Interactive electronic communication were delivered between the patient and the provider or between multiple providers in either synchronous or asynchronous settings for the provision of health care services or consultation.	The control group only received routine treatment and care from the clinic.
Owolabi et al [30], 2019	SMS text messages (6 mo)	Participants in the intervention arm received daily educational SMS text messages on diabetes for 6 months. In addition, the intervention group received the text at an agreed time of the day, according to their needs, care plan, and goals.	The control groups proceeded with their usual care including all medical visits, tests, and diabetes support at the clinic.
Sarfo et al [31], 2019	SMS text messages (9 mo)	Patients received a Bluetooth blood pressure device and smartphone with an app for monitoring blood pressure measurements and medication intake under nurse guidance for 3 months. Participants also received motivational and support messages, advice on lifestyle behaviors like diets, physical activity, smoking cessation, and medication and appointment reminders.	The control arm received only the usual care.

**Table 4.** Study outcome for blood pressure.

Studies	Sample size, N	Age (years), mean (SD)	Intervention, mean (SD)		Control, mean (SD)	
			Systolic blood pressure (mm Hg)	Diastolic blood pressure (mm Hg)	Systolic blood pressure (mm Hg)	Diastolic blood pressure (mm Hg)
Adjei et al [26], 2015	200	47.6 (9.1)	122.9 (18.3)	71.3 (8.5)	124.8 (4.2)	72.3 (9.7)
Asante et al [27], 2020	60	55.1 (10.9)	134 (27.4)	85.2 (17)	150.9 (24.9)	87.3 (12.9)
Bobrow et al [28], 2016^a^	1372	54.3 (11.5)	132.7 (17.5)	—^b^	134.3 (17.3)	—
Bobrow et al [28], 2016^c^	1372	54.3 (11.5)	132.1 (16.6)	—	134.3 (17.3)	—
Kingue et al [29], 2013	268	59.9 (10.4)	169.2 (27.9)	100.4 (18.3)	160.8 (23.7)	95.2 (14.8)
Owolabi et al [30], 2019	216	60.6 (11.6)	144.3 (21.2)	82.3 (10.3)	146.3 (23.8)	82.8 (15.1)
Sarfo et al [31], 2019	60	55 (13)	141.3 (30.3)	91.4 (18.0)	146.3 (22.5)	89.6 (12.9)

a Interactive intervention group vs control.

b Not available.

c Information only intervention group vs control.

**Table 5.** Study outcome for hemoglobin A_1c_ (HbA_1c_).

Studies	Sample size, N	Age (years), mean (SD)	Intervention HbA_1c_ (%), mean (SD)	Control HbA_1c_ (%), mean (SD)
Abaza and Marschollek [25], 2017 (baseline)	73	51.2 (8.7)	9.8 (2.5)	9.5 (2.8)
Abaza and Marschollek [25], 2017 (end point)	73	51.2 (8.7)	8.7 (2.0)	8.8 (2.4)
Asante et al [27], 2020	60	55.1 (10.9)	9.5 (2.0)	9.1 (1.7)

### Meta-Analysis of the Effects on Primary Outcomes

A total of 7 studies, 5 for blood pressure [26-29,31] and 2 for HbA_1c_ [25,27], were included in the meta-analysis.

#### Systolic Blood Pressure

**Figure 2.**
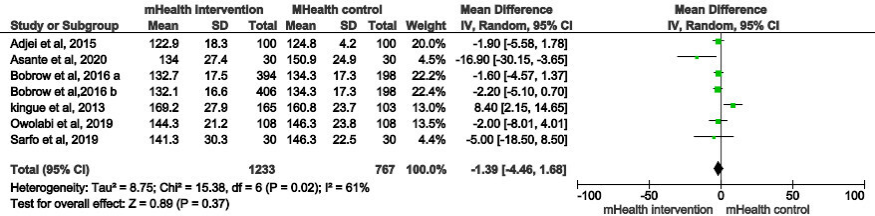
Forest plot of the difference in systolic blood pressure between the mHealth intervention group and control group in 6 studies [26-31]. Bobrow et al [28]: (A) interactive intervention group vs control; (B) information only intervention group vs control. mHealth: mobile health.

**Figure 3.**
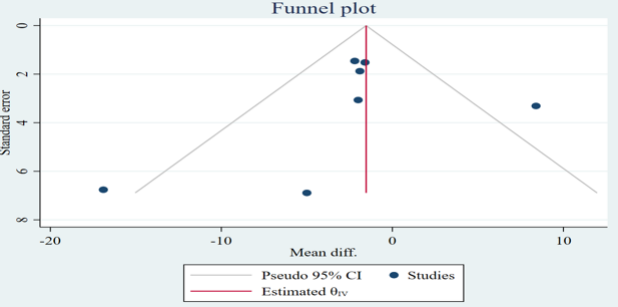
Funnel plot of the difference in systolic blood pressure between the mobile health intervention group and control group. diff.: difference.

As shown in Figure 2, one study had more than one intervention group with the same outcome measured [28]; therefore, 7 interventions are shown in the forest plot of systolic blood pressure, and the estimated WMD of systolic blood pressure between intervention and control groups was not statistically significant at −1.39 mm Hg (95% CI −4.46 to 1.68; *P*=.37; *I*^2^=61%). No significant publication bias was detected visually by the funnel plot (Figure 3) or statistically by Begg (*P*=.30) and Egger (*P*=.10) tests.

#### Diastolic Blood Pressure

**Figure 4.**
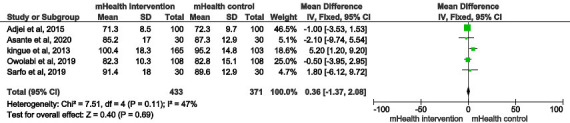
Forest plot of the difference in diastolic blood pressure between the mHealth intervention group and control group in 5 studies [26,27,29-31]. mHealth: mobile health.

**Figure 5.**
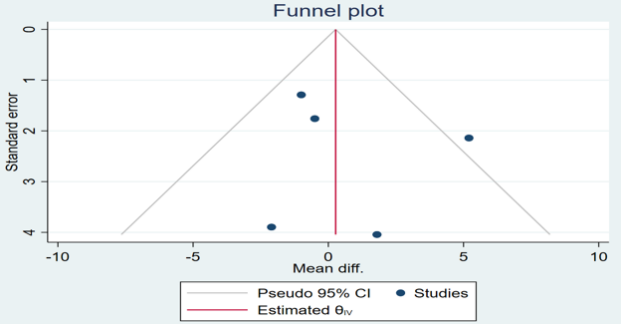
Funnel plot of the difference in diastolic blood pressure between the mobile health intervention group and control group. diff.: difference.

There was no statistically significant difference in diastolic blood pressure (0.36 mm Hg, 95% CI −1.37 to 2.08; *P*=.69; *I*^*2*^=47%) between the intervention and control groups (Figure 4). No significant publication bias was detected visually by the funnel plot (Figure 5) or statistically by Begg (*P*=.65) and Egger (*P*=.81) tests.

#### Hemoglobin A_1c_

**Figure 6.**
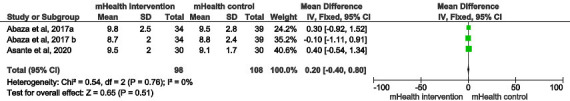
Forest plot of the difference in hemoglobin A_1c_ between the mHealth intervention group and control group in 2 studies [25,27]. Abaza et al [25]: (A) baseline measurement; (B) end point measurement. mHealth: mobile health.

**Figure 7.**
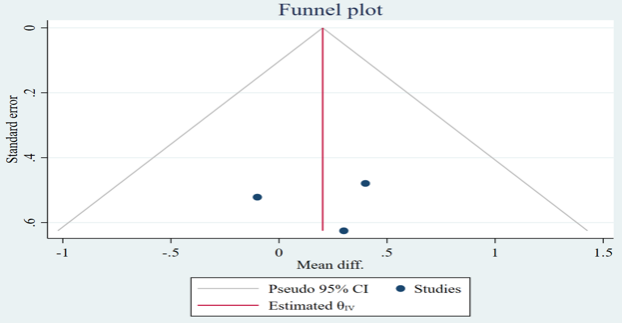
Forest plot of the difference in hemoglobin A_1c_ between the mobile health intervention group and control group. diff.: difference.

Additionally, as shown in Figure 6, one study had more than one intervention group with the same outcome measured [25]; hence, 3 interventions were shown in the forest plot of HbA_1c_, and the mHealth intervention had no significant lowering effects on HbA_1c_ levels among patients with diabetes in the pooled meta-analysis at 0.20 mmol/mol (95% CI −0.40 to 0.80; *P*=.51; *I*^2^=0%). No significant publication bias was detected visually by the funnel plot (Figure 7) or statistically by Begg (*P*=.96) and Egger (*P*=.10) tests.

### Sensitivity Analyses

**Figure 8.**
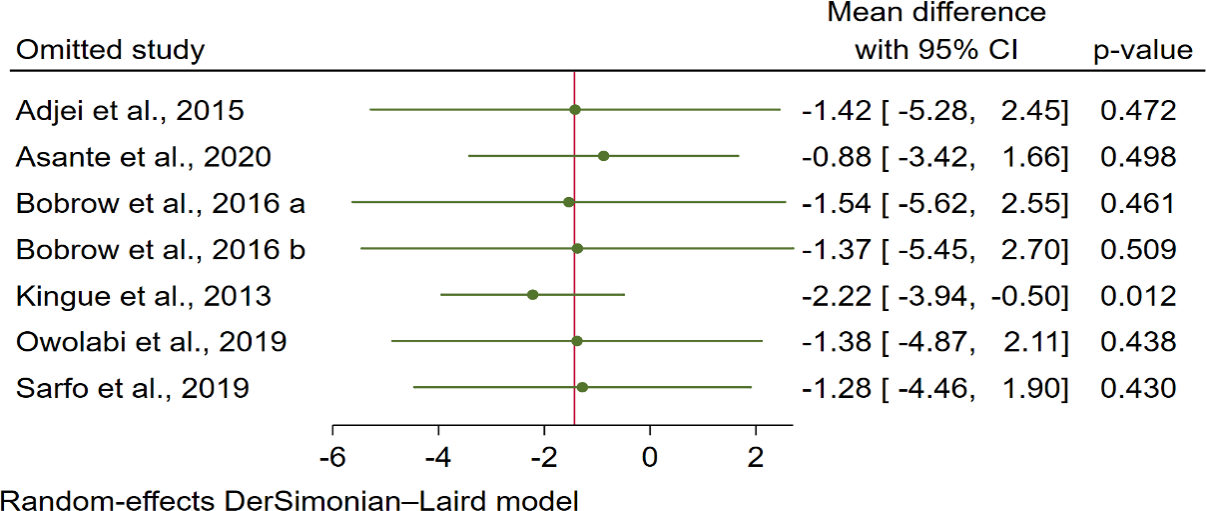
Leave-one-out forest plot for systolic blood pressure in 6 studies [26-31]. Bobrow et al [28]: (A) interactive intervention group vs control; (B) information only intervention group vs control.

**Figure 9.**
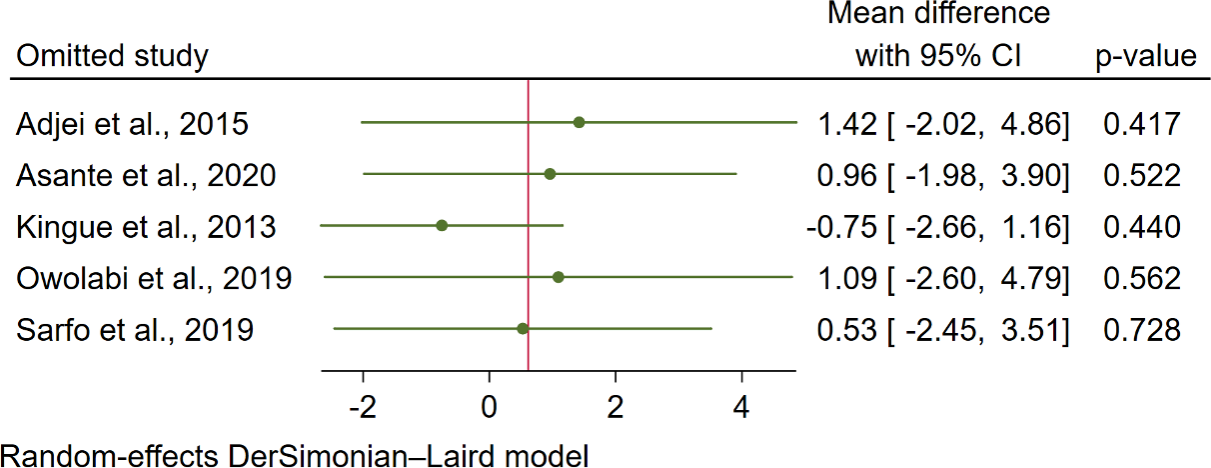
Leave-one-out forest plot for diastolic blood pressure in 5 studies [26,27,29-31].

**Figure 10.**
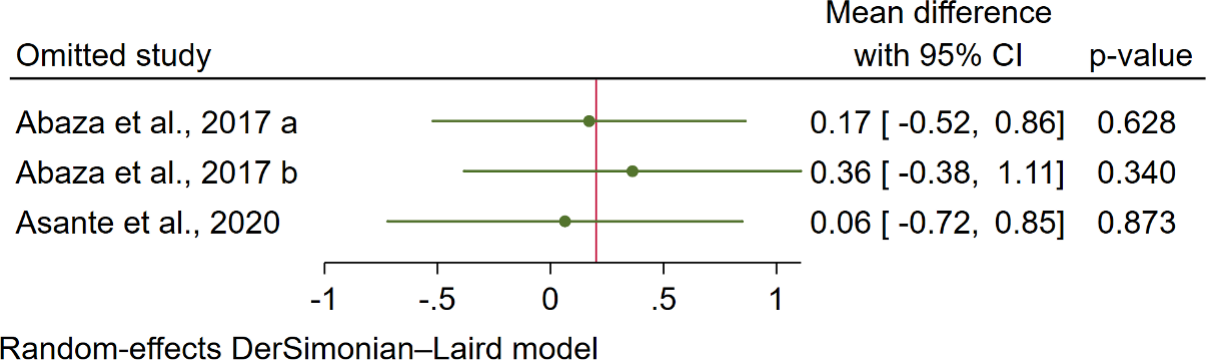
Leave-one-out forest plot for hemoglobin A_1c_ in 2 studies [25,27]. Abaza et al [25]: (A) baseline measurement; (B) end point measurement.

Sensitivity analyses were conducted using the leave-one-out method. For systolic blood pressure, the Kingue et al [29] study had an impact on the WMD, with the pooled WMD being statistically significant after the exclusion of the Kingue et al [29] study (−2.22, 95% CI −3.94 to −0.60; *P*=.01; Figure 8). For diastolic blood pressure and HbA_1c_, the exclusion of each of the studies rendered the WMD nonsignificant (Figures 9 and 10).

## Discussion

### Discussion of the Key Findings

This systematic review and meta-analysis identified 7 RCT studies that assessed the effectiveness of mHealth interventions on blood pressure and glycemic control among patients with hypertension and diabetes in Africa. In this review, the effectiveness of mHealth interventions on blood pressure and glycemic control among patients with hypertension and diabetes in Africa did not show conclusive evidence.

### Systolic Blood Pressure Control

For systolic blood pressure, we observed a reduction after the mHealth intervention compared to usual care by an average of 1.39 mm Hg; however, it was not statistically significant. After conducting a leave-one-out analysis, a study by Kingue et al [29] had an impact on the WMD, and the exclusion of this study resulted in a pooled WMD reduction of 2.22 mm Hg in systolic blood pressure. This finding is consistent with previous RCT studies [20,34-36] that examined mHealth interventions on systolic blood pressure control and showed that mHealth interventions reduced systolic blood pressure by 10.4 mm Hg [20], 5.5 mm Hg [34], 3 mm Hg [35], and 3.9 mm Hg [36], respectively. In contrast, a study performed by Rubinstein et al [13] reported that the mHealth intervention did not reduce systolic blood pressure compared with usual care. This discrepancy could be explained by the relatively small sample number of studies included in this review. Another reason could be due to the different study populations, interventions, ages, and medications used.

### Diastolic Blood Pressure Control

For diastolic blood pressure, our study observed no lowering effect of the mHealth intervention, which reduced by an average of 0.36 mm Hg, which is inconsistent with studies performed by Lu et al [36] and Zhang et al [20] who reported a reduction of 2.2 and 4.8 mm Hg, respectively, after the mHealth intervention compared to usual care. The disparity is that the previous studies [20,36] were conducted among patients with stroke and heart failure with a more complicated pathogenesis of hypertension, which might have resulted in the observed significant decrease in diastolic blood pressure control in this study. In patients with stroke, lower blood pressure might be achieved with strict treatment targets that also lead to a controlled condition. In previous studies, they noted a significant net reduction in body weight and intake of high-fat and high-sugar foods after the intervention [20,36]. Despite no significant findings on diastolic blood pressure control after mHealth interventions, the study by Rubinstein et al [13] reported that each 1 mm Hg decrease in diastolic blood pressure is associated with a 7% decrease in mortality from stroke and ischemic heart disease. Thus, the mHealth intervention may still be a measure worth considering for reducing blood pressure.

### Glycemic Control

For glycemic control, the meta-analysis results showed no improvement after the mHealth interventions. Our study contradicts previous studies by Mao et al [35], Moattari et al [37], Kitsiou et al [4], and Huang et al [38] who found significant improvements in glycemic control following mHealth interventions among patients with diabetes. These studies have reported that patients and health care professionals who communicated by SMS text messages, telephone calls, and even electronic reminders or web servers reported greater improvement in HbA_1c_ outcomes compared with usual care [4,35,37,38]. Thus, patients with poorly controlled diabetes might benefit more from using mHealth, therefore more clinical trials are needed to confirm these findings. The Adjei and Marschollek [25] study in Ghana, despite not reporting HbA_1c_, saw a substantial reduction in fasting plasma glucose of −1.6 mmol/L. Although evidence is scarce about the effect of mHealth interventions on the management of patients with diabetes, the difference could be attributed to the better care the system generates from the health providers. Another reason is that long-term interventions likely result in more significant changes in glycemic control than short-term mHealth interventions.

### Strengths and Limitations

To the best of our knowledge, this review was the first that assessed the effectiveness of mHealth interventions in diabetes and hypertension management in Africa. Quality appraisal suggests that the quality of the included studies was good. Additionally, the included studies show no publication bias. However, there are limitations to acknowledge. Despite a thorough search, the number of included studies was relatively small, signifying that using mHealth interventions in Africa on patients with hypertension and diabetes remains an emerging area. This review may not be able to capture some significant effects due to the small samples in the included studies. Given the above limitations, future studies with larger samples are needed to validate our findings.

### Conclusion

Our study showed no conclusive evidence on the effect of mHealth interventions on systolic blood pressure, diastolic blood pressure, or glycemic control. However, the sample sizes of the included studies were small; therefore, there is a need for larger RCT studies to confirm these findings.

## Supplementary material

10.2196/43742 Multimedia Appendix 1 PRISMA (Preferred Reporting Items for Systematic Reviews and Meta-Analyses) checklist.

10.2196/43742 Multimedia Appendix 2 Supplementary keywords used in the search.

## Abbreviations

HbA_1c_: hemoglobin A_1c_
MeSH: Medical Subject Headings
mHealth: mobile health
PRISMA: Preferred Reporting Items for Systematic Reviews and Meta-Analyses
RCT: randomized controlled trial
WMD: weighted mean difference
