# Supplementary material for: Assessing the Effectiveness of mHealth Interventions for Diabetes and Hypertension Management in Africa: Systematic Review and Meta-Analysis
Source: JMIR Mhealth Uhealth. 2023 Aug 29;11:e43742. doi: 10.2196/43742 (PMC10477453; doi:10.2196/43742)
Supplement: Multimedia Appendix 1 [file mhealth-v11-e43742-s001.pdf]

PRISMA 2020 Checklist

Section and Topic	Item #	Checklist item	Location where item is reported
**TITLE**			
Title	1	**Assessing the effectiveness of mobile health interventions (mHealth) in diabetes and hypertension management in Africa – a systematic review and meta-analysis**	
**ABSTRACT**			
Abstract	2	**Background:** Mobile Health (mHealth) interventions are effective in improving chronic disease management, mainly in high-income countries. However, much less is known about the efficacy of mobile health interventions for the reduction of cardiovascular risk factors including hypertension and diabetes, which are rapidly increasing in low- and middle-income countries.<br>**Objective:** This study aimed to assess the efficacy of mobile health interventions regarding managing diabetes and hypertension patients in Africa.<br><br>**Methods:** We searched PubMed, Cochrane Library, Google Scholar, African Journals Online and Web of Science for relevant studies published from inception to July 2022. The main outcomes of interest were changes in glycated haemoglobin A1c (HbA1c) levels, systolic blood pressure, and diastolic blood pressure. The random or fixed effect model was used for the meta-analysis, and the I^2 statistic was used to gauge study heterogeneity. The Z-test and p-values were used to evaluate the effect of mHealth interventions on HbA1c and blood pressure levels.<br>**Results:** This review included 7 studies [7 randomized controlled trials (RCTs)] with a total of 2,249 participants. Two studies assessed the effect of mHealth on glycemic control, and five studies assessed the effect of mHealth on blood pressure control. The use of mHealth intervention was not associated with significant reductions in HbA1c levels (weighted mean difference [WMD] 0.20, 95% CI -0.40 to 0.80, *P*=.51) among diabetic patients and systolic blood pressure (WMD -1.39, 95% CI -4.46 to 1.68, *P*=.37) and diastolic blood pressure (WMD 0.36, 95% CI -1.37 to 2.05, *P*=.69) among hypertensive patients.After conducting a sensitivity analyses using the leave- one- out method Kingue et al study had an impact on the intervention, resulted in 2mmHg reduction in systolic blood pressure (WMD -2.22, 95% CI, -3.94, to -0.60, *P=0.012*) but was non-significant for diastolic blood pressure and HbA1c levels after omitting studies.<br>**Conclusions:** Our review provided no conclusive evidence for the effectiveness of mHealth interventions in reducing systolic blood pressure, diastolic and gycemic in African among persons with diabetes and hypertension. In order to confirm these findings, larger RCT studies are required	
**INTRODUCTION**			
Rationale	3	Although the data on the efficacy of mHealth in the management of diabetes and hypertension in Africa are limited and have not yet been systematically evaluated. Therefore, the present systematic review assessed the effectiveness of mHealth interventions on blood pressure control among hypertension patients and glycemic control among diabetes patients in Africa. The findings of this paper will provide guidance to improve the adoption of mHealth for the management of diabetes and hypertension in Africa countries	
Objectives	4	This study aimed to assess the efficacy of mobile health interventions regarding managing diabetes and hypertension patients in Africa.	
**METHODS**			
Eligibility criteria	5	**Inclusion Criteria**<br><br>We included studies that met the following criteria: (1) hypertension and diabetes patients aged 18 years and above; (2) patients who received	

Section and Topic	Item #	Checklist item	Location where item is reported
		treatment at a selected healthcare setting; (3) the intervention included a mHealth component; (4) results included target values of glycated haemoglobin (HBA1c), systolic blood pressure and diastolic blood pressure; (5) studies were randomized controlled trials; and (6) articles were written in English. (7) Studies conducted in hospitals and primary health centres were eligible.<br>**Exclusion Criteria**<br>We excluded studies in which (1) full text articles were not available after attempts to contact the author (2) the research participants were pregnant women and or specific patient populations e.g., cancer patients; (3) the results did not describe primary outcomes; (4) the primary intervention did not use mHealth devices; and (5) unpublished manuscripts and conference abstracts.	
Information sources	6	PubMed, Cochrane Library, Google Scholar, African Journals Online and Web of Science were searched for relevant studies published from inception to July 2022 assisted by a clinical librarian. The full search strategies, the common Medical Subject Headings and search terms used across databases are available in Supplementary Table 1. The reference lists of the included studies were hand-searched to identify additional relevant studies.	
Search strategy	7	**Google Scholar**<br>"Treatment Outcome" OR efficacy OR potency AND "Telemedicine" OR "mobile device$" OR "mobile technology" OR "mobile app" OR "mobile app$" OR "mobile health care" AND "Early Medical Intervention" OR mediation OR interference AND "Diabetes Mellitus" OR "high blood glucose" OR "impaired blood glucose" AND "Hypertension" OR "high blood pressure" OR "blood pressure" AND "Disease Management" OR administration AND "Africa" OR "Africa countries"<br>**PubMed**<br>((((((''Telemedicine" OR "mobile device$" OR "mobile technolog*OR "mobile app$" OR "mobile app$" OR "mobile health care" AND ("methods" OR "mediat*"OR "interfer*"AND ("Diabetes Mellitus" OR" high blood glucose" OR "impaired blood glucose" OR ("hypertension" OR "high blood pressure" OR "blood pressure" AND ("Disease Management" OR "administration"AND ("Africa" OR "Africa countries"))<br>**Cochrane**<br>Diabetes OR Hypertension OR DM OR HPT OR t2dm OR t1dm OR prediabetes OR prediabetic OR prediabetes OR prediabetes OR impaired glucose OR high blood pressure OR BP OR HTN in Title Abstract Keyword AND mHealth OR mobile health OR Health OR Mobile OR Telehealth OR ehealth OR e-health in Title Abstract Keyword AND High blood pressure OR systolic blood pressure OR diastolic blood pressure OR SBP OR DBP OR glycemic OR blood sugar OR glucose OR diabetes OR hypertension OR diabetic OR hypertensive in Title Abstract Keyword AND Africa OR Northern African OR Southern Africa OR Eastern Africa OR Western Africa OR central Africa in Title Abstract Keyword<br>**Web of Science**	

PRISMA 2020 Checklist

Section and Topic	Item #	Checklist item	Location where item is reported
		Effectiveness AND "Diabetes OR Hypertension" AND Management AND "Africa Countries"<br>**Africa journals online**<br>Effectiveness AND "Diabetes OR Hypertension" AND Management AND Africa	
Selection process	8	Two independent authors (PA and KA) manually assessed and screened studies for both the titles and abstracts and full-text articles using an Excel sheet. Disagreements were resolved by consensus by a third author (CA) as necessary. This was performed in three stages as follows. First, PA screened the titles of all papers to determine their relevance. KA performed a cross-check of the title screening by screening 20% of titles excluded by the first reviewer, and it was confirmed that none of the titles screened by the second reviewer met the inclusion criteria. Second, abstracts of the papers selected after the title screening stage were again screened by (PA and KA) following the same procedure as described in step one. Finally, full texts of potentially relevant papers were retrieved and evaluated by (PA and KA) independently to ascertain their relevance and usefulness to the review. Disagreements were settled through dialogue with (CA) to reach an agreement. Duplicates were also identified using Endnote reference manager (version x9).	
Data collection process	9	Two authors (PA and KA) independently extracted the following study characteristics from each included article using a tested extraction form: first author, year of publication, mean age, the country where the study was conducted, the participant (diabetic or hypertensive patients), mHealth location (primary care settings, hospital, clinics, etc.), condition (diabetes/hypertension), sample size, mHealth intervention, study design and outcome of the intervention(primary outcomes HbA1c, SBP, and DBP)	
Data items	10a	Out of interest were changes in glycated haemoglobin A1c (HbA1c) levels, systolic blood pressure, and diastolic blood pressure	
	10b	first author, year of publication, mean age, the country where the study was conducted, the participant (diabetic or hypertensive patients), mHealth location (primary care settings, hospital, clinics, etc.), condition (diabetes/hypertension), sample size, mHealth intervention, study design and outcome of the intervention(primary outcomesglycated haemoglobin A1c (HbA1c) levels systolic blood pressure and diastolic blood pressure<br>)	
Study risk of bias assessment	11	The quality of each study was assessed using a 28-point scoring system as adopted from the Downs and Black checklist [25]. The included studies focused on the following items for assessment: Items 1 through 10 evaluated whether the information provided was adequate for the reader to make an objective assessment of the study's findings; Items 11 through 13 evaluated external validity, which examined the extent to which study findings could be applied to the population from which the study subjects were drawn; Items 14 through 20 assessed possible bias, which focused on biases in the assessment of the intervention and the result; Items 21 through 26 assessed confounding, which focused on biases in the research participants' selection. To determine if neutral research results may be the result of chance or insufficient power, item 27 evaluated the study's power	
Effect measures	12	The data for primary outcomes (glycated haemoglobin A1c (HbA1c) levels, systolic blood pressure, and diastolic blood pressure) were analyzed	

Section and Topic	Item #	Checklist item	Location where item is reported
		separately using random or fixed effects models with a weighted mean difference (WMD) in Review Manager (RevMan; version 5.4, The Cochrane Collaboration, 2020) [26]. The I^2 statistic was calculated to measure the percentage of variation across trials due to heterogeneity, with values of <50% and ≥50% indicating low and high levels of heterogeneity, respectively. The WMD in blood pressure and HbA1c between the intervention and control Z tests were used to compare groups, and *P* values less than 0.05 were regarded as statistically significant. We checked publication bias subjectively by funnel plot and objectively by Begg's and Egger's tests using STATA version 16. Begg's and Egger's tests with $P <$ .05 were considered significant publication bias.	
Synthesis methods	13a	PA and KA) independently extracted the following study characteristics from each included article using a tested extraction form: first author, year of publication, mean age, the country where the study was conducted, the participant (diabetic or hypertensive patients), mHealth location (primary care settings, hospital, clinics, etc.), condition (diabetes/hypertension), sample size, mHealth intervention, study design and outcome of the intervention.	
	13b	Describe any methods required to prepare the data for presentation or synthesis, such as handling of missing summary statistics, or data conversions.	
	13c	Describe any methods used to tabulate or visually display results of individual studies and syntheses.	
	13d	(glycated haemoglobin A1c (HbA1c) levels, systolic blood pressure, and diastolic blood pressure) were analyzed separately using random or fixed effects models with a weighted mean difference (WMD) in Review Manager (RevMan; version 5.4, The Cochrane Collaboration, 2020) [26]. The I^2 statistic was calculated to measure the percentage of variation across trials due to heterogeneity, with values of <50% and ≥50% indicating low and high levels of heterogeneity, respectively. The WMD in blood pressure and HbA1c between the intervention and control Z tests were used to compare groups, and *P* values less than 0.05 were regarded as statistically significant. We checked publication bias subjectively by funnel plot and objectively by Begg's and Egger's tests using STATA version 16. Begg's and Egger's tests with $P <$ .05 were considered significant publication bias.	
	13e	The Random effect model was used to account for heterogeneity	
	13f	Sensitivity analyses were conducted using the leave- one- out method in stata 17	
Reporting bias	14	using a 28-point scoring system as adopted from the Downs and Black checklist, we use that to assess the quality of	

Section and Topic	Item #	Checklist item	Location where item is reported
assessment		each study	
Certainty assessment	15	N/A	
**RESULTS**			
Study selection	16a	Records identified through database searching<br>Google Scholar n=2210<br>PubMed n=460<br>Cochrane Library n=200<br>Web of Science n=15<br>Africa Journal Online n=20<br>(n=2905), Additional records identified through other sources n=(3). Records after duplicates were removed (n=2880). Records screened (n=2880).Full-text articles assessed for eligibility (146)<br>Records excluded (n=2734)<br>Studies included in qualitative synthesis (n=16)<br>Studies included in quantitative synthesis (meta-analysis; n=7)<br>Records excluded (n=130)	
	16b	Full-text articles excluded with reasons (n=9)<br><br>Conference articles (n=2)<br>Protocol (n=1)<br>Qualitative studies (n=4)<br>No control reported (1)<br>Cohort study (1)	
Study characteristics	17	Studies included in quantitative synthesis (meta-analysis; n=7) (1) hypertension and diabetes patients aged 18 years and above; (2) patients who received treatment at a selected healthcare setting; (3) the intervention included a mHealth component; (4) results included target values of glycated haemoglobin (HBA1c), systolic blood pressure and diastolic blood pressure; (5) studies were randomized controlled trials; and (6) articles were written in English. (7) Studies conducted in hospitals and primary health centres were eligible.	
Risk of bias in studies	18	(see nested table below)	

Authors, year of publication	Information based on	External validity	Potential bias (14-20)	Confounding (21- 26)	Power of study	Total score (maximum	Quality as per the cut-

Section and Topic	Item #	Checklist item	Location where item is reported
		(table continued below)	

	study findings (1-10)	(11-13)			(27)	score =27)	off described
Abaza et al., [32]	10	3	6	5	0	24	Good
Adjei et al., [31]	10	3	5	5	0	23	Good
Asante et al., [30]	10	3	4	5	0	22	Good
Bobrow et al., [28]	10	3	5	6	0	24	Good
Kingue et al., [29]	10	3	5	6	0	24	Good
Owolabi et al., [33]	10	3	6	5	0	24	Good
Sarfo et al., [27]	10	3	6	6	0	25	Good

Results of individual studies | 19

Table 4 Study intervention and control description

Study	Intervention type	Intervention group	Control group
Abaza et al., [32]	SMS	Patients received daily messages and weekly reminders addressing various diabetes care categories.	The control group did not receive SMS messages but received paper-based educational material.
Adjei et al., [31]	Electronic reminders (webserver)	The intervention group was given electronic reminders for their clinical appointments and their physicians were prompted with abnormal laboratory results for six months.	Patients received only the usual care.
Asante et al., [30]	Mobile phone calls	Mobile phone call intervention delivered by nurses in addition to care as usual over 12 weeks. The intervention group received up to 16 mobile phone calls (mean duration = 12 minutes) from a diabetes specialist nurse in addition to their care as usual.	The control group received only care as usual.
Bobrow et al., [28]	SMS	SMS text messages were delivered automatically via an open-source web-based electronic medical	The usual care group continued to receive care

Section and Topic	Item #	Checklist item	Location where item is reported

		record system. Messages were sent for one year from enrolment. Blood pressure measurements were collected from participants as they attended their routine clinic visits. Delivery of SMS text messages was automatically tracked and if undelivered a research assistant, blinded to group allocation, would contact the number of a friend or relative to obtain a new mobile phone number	from the clinic and some form of written information about hypertension and healthy living but no Personalized SMS text messages were sent.
Kingue et al., [29]	Mobile phone calls	Interactive electronic communication between the patient and the provider or between multiple providers in either synchronous or asynchronous settings for the provision of health care services or consultation	Only received routine treatment and care from the clinic.
Owolabi et al., [33]	SMS	Participants in the intervention arm received daily educational text messages on diabetes for six months. In addition, the intervention group received the SMS at an agreed time of the day, according to their needs, care plan and goals.	The control groups proceeded with their usual care including all medical visits, tests and diabetes support at the clinic.
Sarfo et al., [27]	SMS	Patients received a Blue-toothed blood pressure device and smartphone with an App for monitoring blood pressure measurements and medication intake under nurse guidance for three months. Participants also received motivational and support messages, advice on lifestyle behaviours like diets, physical activity, smoking cessation, medication and appointment reminders.	The control arm received only the usual care.

Section and Topic	Item #	Checklist item	Location where item is reported

Table 5 Study outcome for blood pressure

Reference	Intervention type	Duration of Intervention	Intervention		Control	
			Systolic Blood Pressure (mmHg)	Diastolic Blood Pressure(mmHg)	Systolic Blood Pressure (mmHg)	Diastolic Blood Pressure (mmHg)
Adjei et al., [31]	Electronic reminders (webserver)	6 months	122.9(18.3)	71.3(8.5)	124.8(4.2)	72.3(9.7)
Asante et al., [30]	Electronic reminders (webserver)	12weeks	134(27.4)	85.2(17)	150.9(24.9)	87.3(12.9)
Bobrow et al., [28] a	SMS	6months	132.7(17.5)	-	134.3 (17.3)	-
Bobrow et al., [28] b	SMS	12months	132.1(16.6)	-	134.3(17.3)	-
Kingue et al., [29]	Mobile phone communication	24 weeks	169.2(27.9)	100.4 (18.3)	160.8 (23.7)	95.2 (14.8)
Owolabi et al., [33]	SMS	6months	144.3(21.2)	82.3 (10.3)	146.3(23.8)	82.8 (15.1)
Sarfo et al., [27]	SMS	9 months	141.3 (30.3)	91.4 (18.0)	146.3 (22.5)	89.6 (12.9)

SMS: short message service

Table 6 Study outcome for glycated haemoglobin (HbA1c)

Reference	Intervention Type	Duration of intervention	Intervention	Control
Abaza et al., [32] a	SMS	3months	9.8(2.5)	9.5(2.8)
Abaza et al., [32] b	SMS	3months	8.7(2.0)	8.8(2.4)
Asante et al., [30]	Mobile phone calls	3months	9.5(2.0)	9.1(1.7)

Section and Topic	Item #	Checklist item	Location where item is reported
Results of syntheses	20a	For each synthesis, briefly summarise the characteristics and risk of bias among contributing studies.	
	20b		

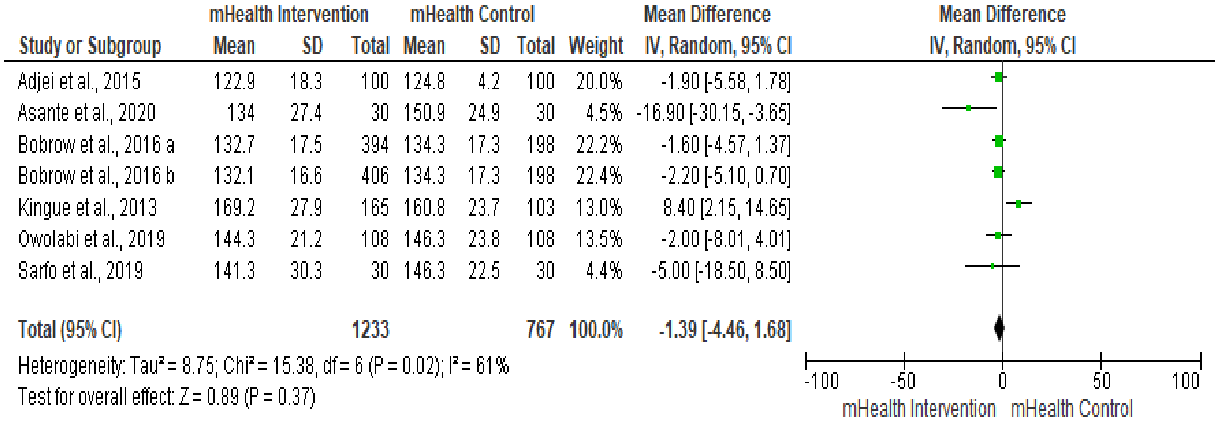

Figure 1 Forest plot of the difference in systolic blood pressure between mHealth intervention and mHealth control

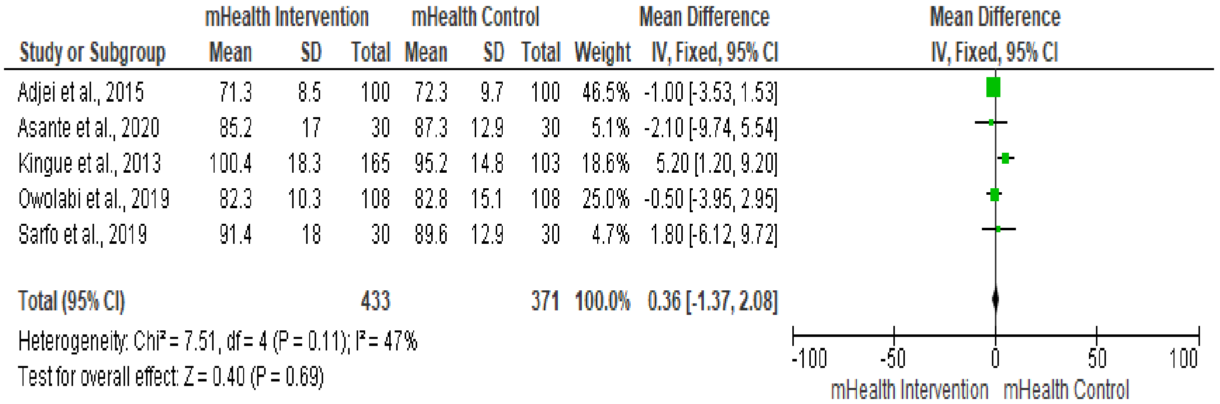

Figure 5 Forest plot of the difference in Diastolic Blood Pressure between mHealth intervention and mHealth control

Section and Topic	Item #	Checklist item	Location where item is reported
		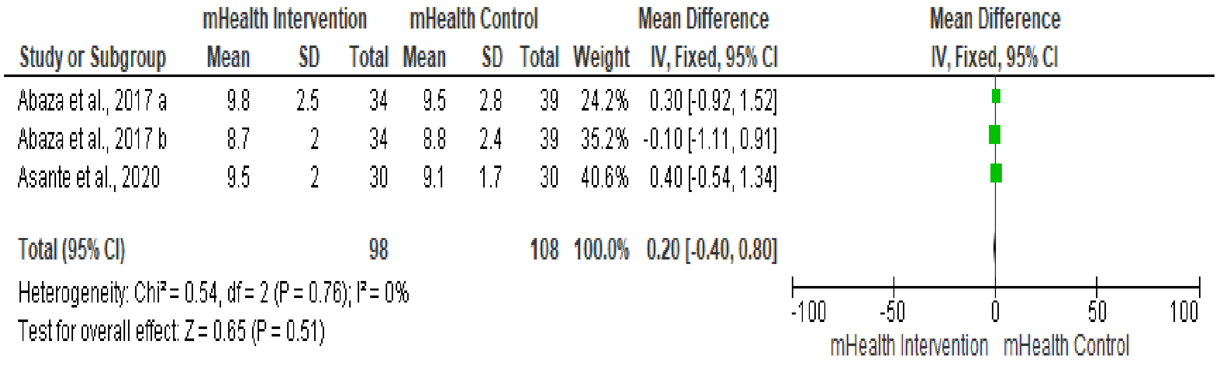 **Figure 8 Forest plot of the difference of HbA1c between mHealth intervention and mHealth control**	
	20c	Present results of all investigations of possible causes of heterogeneity among study results.	
	20d	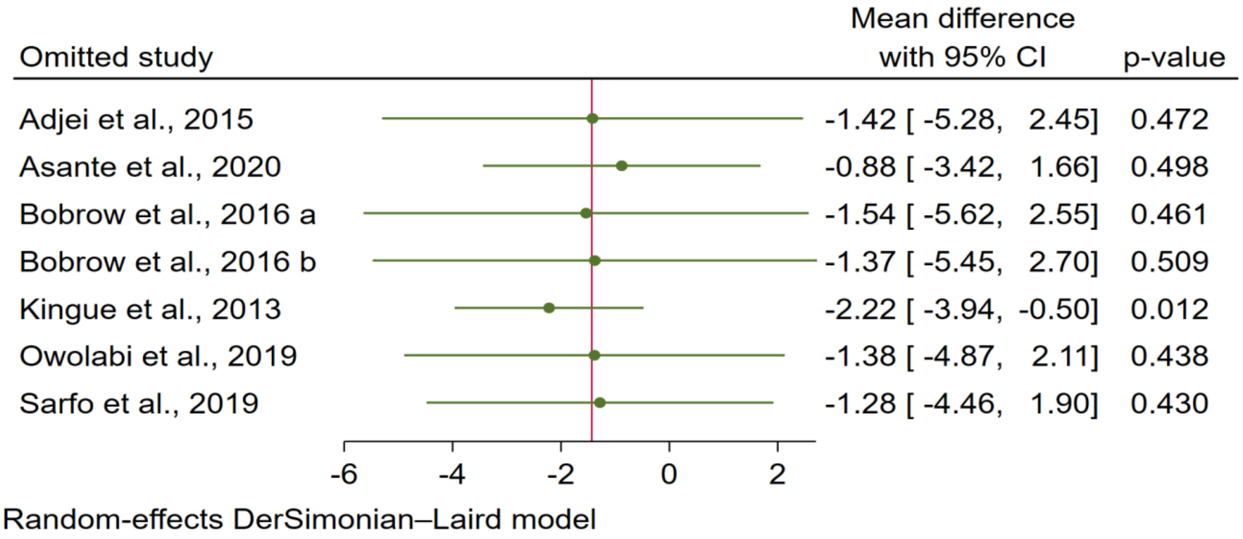 **Fig 4Fig Leave-one-out forest plot Systolic Blood Pressure**	

Section and Topic	Item #	Checklist item	Location where item is reported

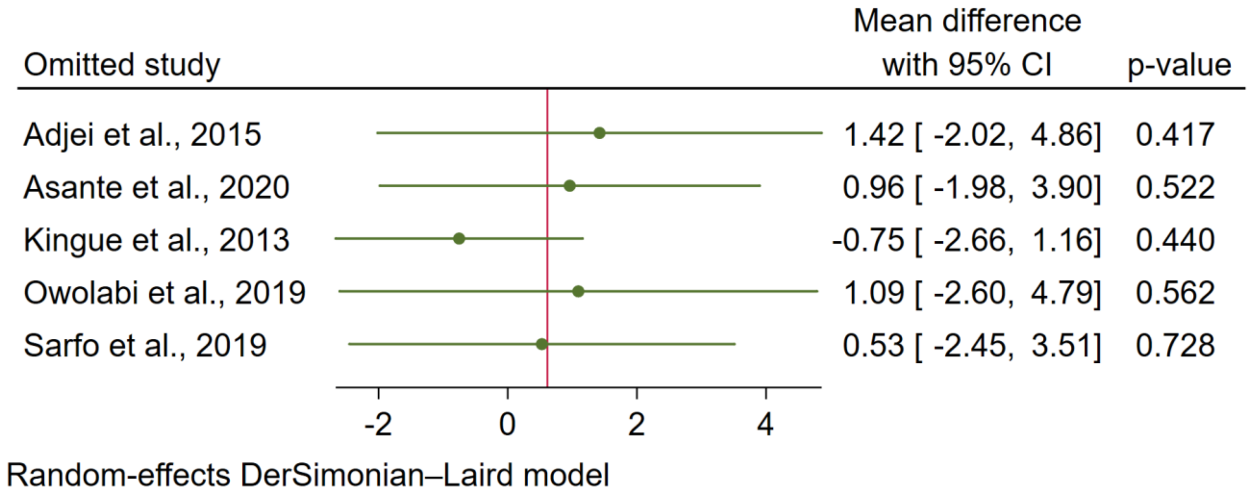

Fig 7: Leave-one-out forest plot Diastolic Blood Pressure

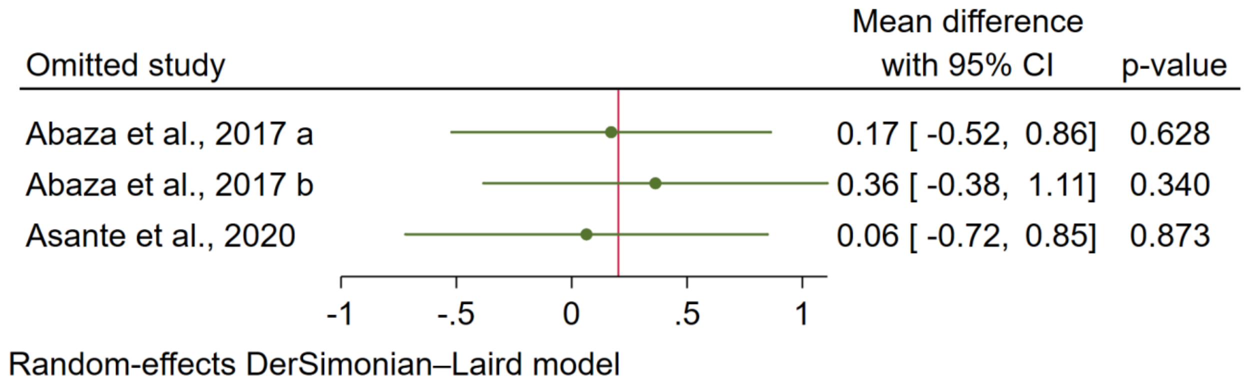

Fig10: Leave-one-out forest plot HBA1c

Reporting biases	21	NA	
Certainty of evidence	22	NA	
**DISCUSSION**			

Section and Topic	Item #	Checklist item	Location where item is reported
Discussion	23a	This systematic review and meta-analysis identified 7 RCT studies that assessed the effectiveness of mHealth interventions on blood pressure and glycemic control among hypertensive and diabetic patients in Africa. In this review, the effectiveness of mHealth interventions on blood pressure and glycemic control among hypertensive and diabetic patients in Africa did not show a conclusive evidence.<br><br>**Systolic blood pressure control**<br>For SBP, we observed a reduction after the mHealth intervention compared to usual care by an average of 1.39 mm Hg, however, it was not statistically significant. After conducting leave- one- out analysis, a study by Kingue et al[29], had an impact on the WMD, and exclusion of this study resulted in pooled WMD of 2.22 mmHg reduction in SBP. This finding is consistent with previous RCT studies [21, 34–36] that examined mHealth interventions on SBP control that show that mHealth interventions reduced SBP by 10.4 mmHg [21], 5.5 mmHg [34], 3 mmHg [35] and 3.9 mmHg [36], respectively. In contrast, a study performed by Rubinstein et al., (2016), reported that the mHealth intervention did not reduce the SBP compared with usual care. This discrepancy could be explained by the relatively small sample number of studies included in this review. Another reason could be due to the different study populations, interventions, ages and medications used.	

Section and Topic	Item #	Checklist item	Location where item is reported
		**Diastolic blood pressure control**<br><br>For DBP, our study observed no lowering effect of mHealth intervention by an average of 0.36 mm Hg, which is inconsistent with studies performed by Lu et al., (2019) and Zhang et al., (2021) who reported a reduction of 2.2 mmHg and 4.8 mmHg after mHealth intervention compared to usual care. The disparity is that the previous studies were conducted among stroke and heart failure patients with a more complicated pathogenesis of hypertension, which might have resulted in the observed significant decrease in DBP control to the present study. In stroke patients, blood pressure lowering might also be achieved with strict treatment targets that also lead to a controlled condition. In their studies, they noted a significant net reduction in body weight and intake of high-fat and high-sugar foods after the intervention [21, 36]. Despite no significant findings on DBP control after mHealth interventions. Several studies have reported that each 2 mm/Hg decrease in SBP or 1 mm/Hg decrease in DBP is associated with a 10% and 7% decrease in mortality from stroke and ischemic heart disease [14, 21, 36, 37]. Thus,mHealth intervention may still be a measure worth considering for reducing blood pressure.<br><br>**Glycated haemoglobin control**<br><br>For glycated haemoglobin (HbA1c) control, the meta-analysis result showed no improvement after mHealth interventions. Our study contradicts previous studies by Mao et al., (2020), Moattari et al., (2013), Kitsiou et al., (2017) and Huang et al., (2019) who found significant improvements in glycemic control following mHealth interventions among diabetes patients. These studies have reported that patients and healthcare professionals who communicated online by text messaging, telephone calls and even electronic reminders or web servers reported greater improvement in HbA1c outcomes compared with usual care [4, 35, 38, 39]. Thus, patients with poorly controlled diabetes might benefit more from using mHealth, therefore more clinical trials are needed to confirm these findings. Adjei et al study in Ghana	

PRISMA 2020 Checklist

Section and Topic	Item #	Checklist item	Location where item is reported
		[31], though did not report on HbA1C, there was a substantial reduction in fasting plasma glucose by −1.6 mmol/L. Although evidence is scarce about the effect of mHealth intervention on the management of patients with diabetes, the difference could be attributed to better care the system generates from the health providers. Another reason is that long-term interventions likely result in more significant changes in HbA1c control than short-term mHealth interventions	
	23b	To the best of our knowledge, this review was the first that assessed the effectiveness of mHealth interventions in diabetes and hypertension management in Africa. Quality appraisal suggests that the quality of the included studies was good. Additionally, the included studies show no publication bias. However, there are limitations to acknowledge. Despite a thorough search, the included studies were relatively small, signifying that using mHealth intervention in Africa on patients with hypertension and diabetes remains an emerging area.	
	23c	To the best of our knowledge, this review was the first that assessed the effectiveness of mHealth interventions in diabetes and hypertension management in Africa	
	23d	This review may not be able to capture some significant effects due to the small studies' samples. Given the above limitations, future studies with large studies are needed to validate our findings.	
**OTHER INFORMATION**			
Registration and protocol	24a	**PROSPERO** CRD42021230642, Pearl Aovare	
	24b	**PROSPERO** CRD42021230642	
	24c	NA	
Support	25	Funding/sponsorship for the research was from the Africa eHealth Foundation. And funder read and approved the final manuscript. All authors had access to the data presented in this paper and accept the responsibility to submit it for publication.	

PRISMA 2020 Checklist

Section and Topic	Item #	Checklist item	Location where item is reported
Competing interests	26	The authors declare no competing interests.	
Availability of data, code and other materials	27	Refer to Study references	

From: Page MJ, McKenzie JE, Bossuyt PM, Boutron I, Hoffmann TC, Mulrow CD, et al. The PRISMA 2020 statement: an updated guideline for reporting systematic reviews. BMJ 2021;372:n71. doi: 10.1136/bmj.n71

For more information, visit: http://www.prisma-statement.org/
