# Supplementary figures and images for: Assessing the Effectiveness of mHealth Interventions for Diabetes and Hypertension Management in Africa: Systematic Review and Meta-Analysis
Source: JMIR Mhealth Uhealth. 2023 Aug 29;11:e43742. doi: 10.2196/43742 (PMC10477453; doi:10.2196/43742)

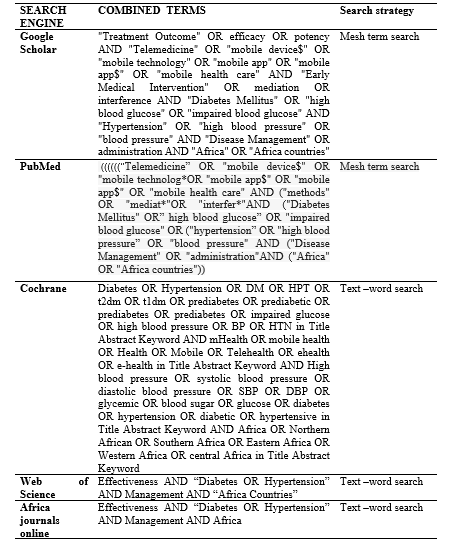

Supplement: Multimedia Appendix 2 [file mhealth-v11-e43742-s002.png]
